# The Dynamic Feature of Macrophage M1/M2 Imbalance Facilitates the Progression of Non-Traumatic Osteonecrosis of the Femoral Head

**DOI:** 10.3389/fbioe.2022.912133

**Published:** 2022-04-27

**Authors:** Zhen Tan, Yan Wang, Yingqi Chen, Youwen Liu, Maoxiao Ma, Zetao Ma, Chao Wang, Hui Zeng, Lixiang Xue, Chen Yue, Deli Wang

**Affiliations:** ^1^ Department of Bone and Joint Surgery, Peking University Shenzhen Hospital, Shenzhen Peking University-The Hong Kong University of Science and Technology Medical Center, Shenzhen, China; ^2^ Center of Basic Medical Research, Institute of Medical Innovation and Research, Peking University Third Hospital, Beijing, China; ^3^ Department of Bone and Joint Surgery, National and Local Joint Engineering Research Center of Orthopaedic Biomaterials, Peking University Shenzhen Hospital, Shenzhen, China; ^4^ Department of Orthopedic, Luoyang Orthopedic Hospital of Henan Province, Orthopedic Hospital of Henan Province, Luoyang, China

**Keywords:** non-traumatic osteonecrosis of the femoral head (NONFH), macrophage, chronical inflammation microenviroment, multiplex immunohistochemistry (mIHC), cytometric bead array (CBA)

## Abstract

Non-traumatic osteonecrosis of the femoral head (NONFH) remains a common refractory disease with poorly understood pathogenesis. Macrophage M1/M2 imbalance and chronic inflammatory microenvironment have been suggested to be closely related to osteonecrosis. Here we describe direct visual evidence for the involvement of dynamic changes in macrophages and the chronic inflammatory microenvironment in human NONFH. Osteonecrosis induces inflammatory responses and macrophage enrichment in the reparative area, and the number of inflammatory cells and macrophages falls during progressive-to end-stage NONFH. Multiplex immunohistochemistry demonstrated that macrophage M1/M2 ratio increased from 3 to 10 during progressive-to end-stage. During the progressive-stage, new blood vessels formed in the reparative area, M2 macrophages accumulated in perivascular (M1/M2 ratio ∼0.05), while M1 macrophages were enriched in avascular areas (M1/M2 ratio ∼12). Furthermore, inflammatory cytokines were detected in synovial fluid and plasma using cytometric bead arrays. Interleukin (IL)-6 and IL-1β were persistently enriched in synovial fluid compared to plasma in patients with NONFH, and this difference was confirmed by immunohistochemistry staining. However, only IL-6 levels in plasma were higher in patients with progressive-stage NONFH than in osteoarthritis. Moreover, fibrosis tissues were observed in the necrotic area in progressive-stage and end-stage NONFH based on Sirius Red staining. Together, these findings indicate that macrophage M1/M2 imbalance facilitates the progression of NONFH, a chronic inflammatory disease characterized by chronic inflammation, osteonecrosis and tissue fibrosis in the local lesion. Inhibiting inflammation, promoting the resolution of inflammation, switching macrophages to an M2 phenotype, or inhibiting their adoption of an M1 phenotype may be useful therapeutic strategies against NONFH.

## Introduction

Non-traumatic osteonecrosis of the femoral head (NONFH) is a chronic, destructive disease affecting younger patients during their working life. NONFH is associated with systemic steroid administration and habitual alcohol use, and it involves progressive bone necrosis that leads to early collapse of the femoral head and substantial loss of hip joint function (Fukushima et al., 2010; Li et al., 2020; Zhu et al., 2020; Cui et al., 2021; Song et al., 2021). Epidemiological studies indicated that over 200,000 cases of osteonecrosis of the femoral head (ONFH) are diagnosed annually in the United State, and approximately eight million people in China have the condition (Petrigliano and Lieberman, 2007; Zhao et al., 2015; Piuzzi et al., 2017; Xu et al., 2020). While dysregulation of fat metabolism, intravascular coagulation, abnormally intraosseous hypertension and arterial vasculitis have been suggested to affect the occurrence and development of NONFH, the precise pathogenesis of NONFH are still poorly understood.

Recently, extensive studies have focused on the key role of inflammation in bone regeneration and bone related disease. Acute inflammation is the first step in bone regeneration and repair (Goodman and Maruyama, 2020; Newman et al., 2021). However, when the endogenous adverse stimuli persist or the normal homeostatic mechanisms cannot resolve the injury, chronic inflammation occurs at the site of injury (Goodman and Maruyama, 2020; Newman et al., 2021). Chronic inflammation is detrimental to all tissues and organs, and it is a major driver of osteonecrosis (Goodman and Maruyama, 2020; Chen et al., 2021). Within the chronic inflammatory microenvironment, the tissue continuously undergoes repair, thus local fibrosis occurs, which hinders the reconstruction of normal bone anatomy and function (Goodman and Maruyama, 2020; Chen et al., 2021). NONFH is essentially a disorder of bone regeneration and repair following steroid or alcohol-induced bone injury. However, which inflammatory cytokines are involved in maintaining the chronic inflammation and promoting tissue fibrosis are unclear, as are the dynamic changes in cytokine levels that occur.

Macrophages are heterogeneous cells that circulate in the blood or are concatenated in different organs and tissues; they constitute the first barrier against disease (Loi et al., 2016; Garcia-Garcia and Martin, 2019; Yang et al., 2019; Goodman and Maruyama, 2020). Among immune cells, macrophages are the vital modulators of inflammation and can be programmed to pro-inflammatory (M1) or anti-inflammatory (M2) phenotypes that allow them to fine-tune the regeneration and repair of tissues (Ono and Takayanagi, 2017; Locati et al., 2020; Newman et al., 2021). Proinflammatory M1 macrophages secrete inflammatory cytokines such as tumor necrosis factor (TNF)-α, interleukin (IL)-6, and IL-1β, which trigger an inflammatory cascade and establish a inflammatory microenvironment. This dominates the acute inflammatory response during the first 3–7 days after bone injury (Wynn and Vannella, 2016; Ono and Takayanagi, 2017). Subsequently, M1 macrophages are reprogrammed to an anti-inflammatory M2 phenotype, and they secrete anti-inflammatory cytokines such as transforming growth factor (TGF)-β and IL-10 to resolve inflammation, help form blood vessels and reconstruct normal bone (Wynn and Vannella, 2016; Ono and Takayanagi, 2017). Before bone remodeling around 21 days after bone injury, the M1 and M2 macrophages complete their “mission” and are no longer detectable in the microenvironment (Schlundt et al., 2018). However, if M1 macrophages fail to shift to an M2 phenotype, macrophage M1/M2 imbalance results, triggering chronic inflammation and inducing tissue fibrosis. These are the main barriers to tissue regeneration and repair (Sica and Mantovani, 2012; Loi et al., 2016; Wynn and Vannella, 2016; Ono and Takayanagi, 2017; Tang et al., 2019). Although M1 macrophages have been detected in an animal model of steroid-induced ONFH (Wu et al., 2015), the distribution of M1 and M2 macrophages as well as the dynamic changes in the M1/M2 balance remain unclear in human NONFH.

In this study, we examined the distribution of inflammatory cells and macrophages, and dynamic changes in the macrophage M1/M2 balance in human NONFH during progressive-stage to end-stage. Furthermore, we explored the enrichment of inflammatory factors in the local lesion, and the resulting degree of tissue fibrosis in the human necrosis femoral head. Finally, we hope, through the human specimens, to elucidate the dynamic changes of macrophage M1/M2 in NONFH, and the role of macrophage M1/M2 imbalance, chronic inflammation microenvironment in NONFH.

## Materials and Methods

### Harvesting and Processing of Human Non-Traumatic Osteonecrosis of the Femoral Head Specimens

This study was approved by the Research Ethics Committee of Peking University Shenzhen Hospital. All patients provided written informed consent to participate in the research. From January 2020 to January 2021, we recruited patients who were diagnosed with NONFH or osteoarthritis and who underwent total hip arthroplasty at the Peking University Shenzhen Hospital. We divided patients with NONFH into those whose disease was attributed to steroids (supraphysiological glucocorticoid therapy, long-term glucocorticoid therapy, other steroid used), alcohol (>400 ml of alcohol per week (Matsuo et al., 1988)) or an idiopathic cause. NONFH was staged based on the Ficat classification of osteonecrosis. In the end, the trial enrolled 90 patients with NONFH, comprising 15 patients each with steroid-induced, alcohol-induced or idiopathic NONFH in Ficat stage III or IV. For comparison, 15 patients with osteoarthritis were also enrolled (patients with cystic heaviness of the subchondral bone were excluded). The patients demographic information were shown in the Supplementary Table S1.

#### Plasma Harvest

Preoperative venous blood was harvested into EDTA anticoagulation tubes and centrifuged at 3,000 rpm for 10 min at 4°C. The plasma supernatant was transferred to a cryopreservation tube and stored in liquid nitrogen for subsequent analysis in the cytometric bead array (see below).

#### Synovial Fluid Harvest

During the operation, synovial fluid was harvested using a 5-ml syringe after cutting the capsule using an electric scalpel. Synovial fluid was transferred to a cryopreservation tube, stored and processed as described for preoperative plasma.

#### Femoral Head Harvest

**FIGURE 1:**
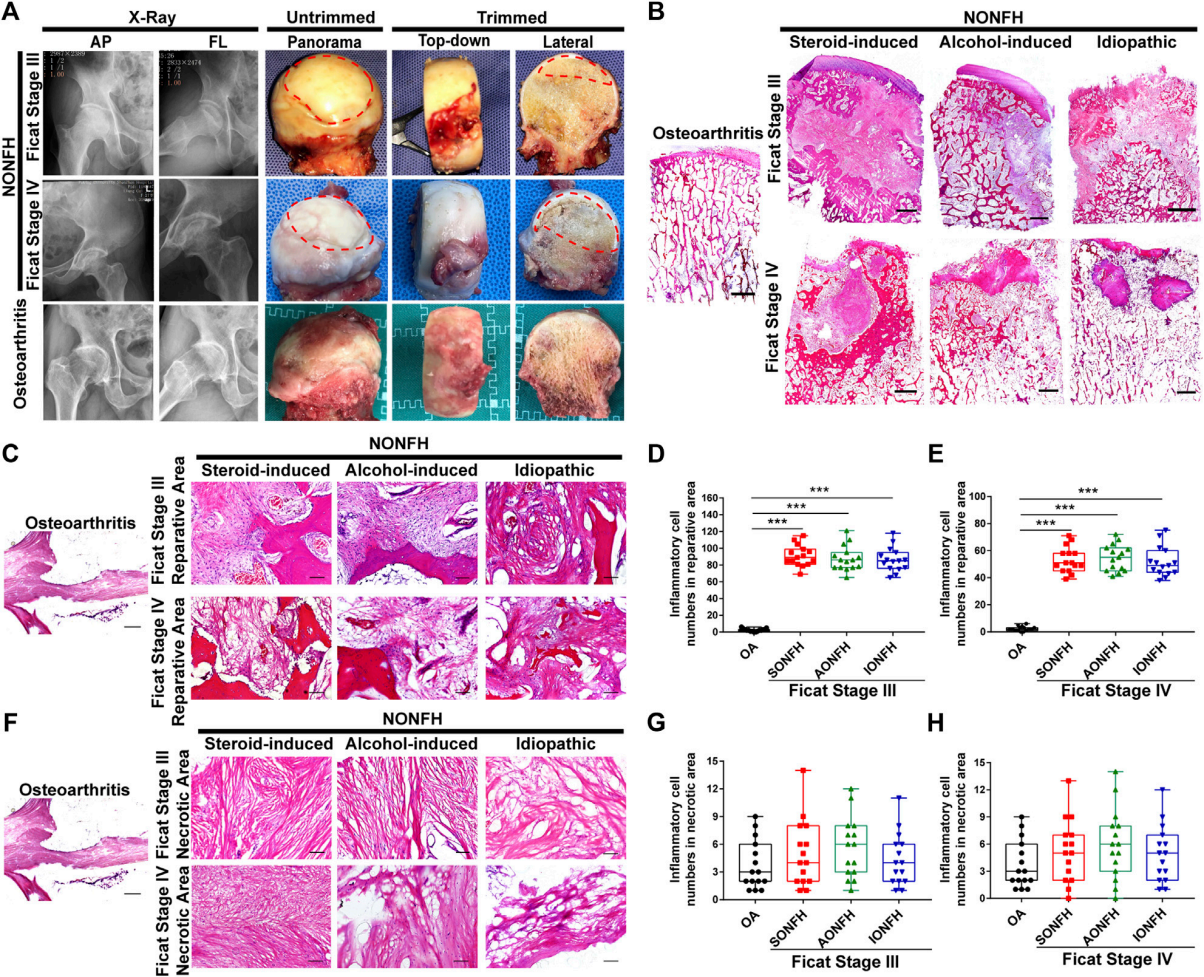
Inflammatory cells were enriched mainly in the reparative area sites of repair and their numbers gradually fell during progressive-stage to end-stage NONFH **(A)** Representative X-ray and femoral heads gross sample pictures from patients with NONFH. AP: anteroposterior, FL: frog-leg lateral **(B)** Representative images of HE-stained sections from patients with different types and stages of NONFH. SONFH, steroid-induced osteonecrosis of the femoral head; AONFH, alcohol-induced osteonecrosis of the femoral head; IONFH, idiopathic osteonecrosis of the femoral head. Scale bar, 2.5 mm **(C–E)** Representative images **(C)** and inflammatory cell quantification **(D,E)** in the reparative area in HE-stained tissues from patients with different types of progressive NONFH. Scale bar, 100 μm **(F–H)** Representative images **(F)** and inflammatory cell quantification **(G,H)** in necrotic areas of HE-stained tissues from patients with different types and stages of NONFH. *p < 0.05; **p < 0.01; ***p < 0.001.

During the operation, the femoral head was collected after resection from the femur. Femoral head samples were cut into coronal sections using an electric bone saw (Figure 1A), fixed in 10% neutral formalin for 1 week, and decalcified for 4–6 weeks in 0.5 m EDTA at 37°C on a shaker (HNY-100B, China Changsha Bayue Instruments, Changsha, Hunan, China). The decalcification solution was replaced every 2 days. Decalcified tissue was embedded into a disposable plastic cassette measuring 25 mm × 32 mm × 5 mm (Citotest Labware, Nantong, Jiangsu, China), covered with optimal cutting temperature compound (OCT; catalog no. EM0197-C-A, Avantik, New Jersey, California, United States of America), snap-frozen on dry ice, then stored at −80°C until subsequent histology.

### Preparation of Bone Tissue Slides

Before cryosectioning, adhesive tape windows (catalog no. 39475214, Leica Microsystems, Nussloch, Germany) and a hand roller (catalog no. 39475218, Leica Microsystems) were pre-chilled in a cryostat at −25°C. The surface of slides (catalog no. 148105W, Citotest, Haimen, Jiangsu, China) were evenly coated with Norland optical adhesive 63 (catalog no. NOA63, Norland Products, New Jersey, Australia). The bone tissue surface was exposed, then the frozen adhesive tape was tightly attached to the surface of the bone tissue using the pre-chilled hand roller. Complete bone tissue slices were carefully cut out, attached to slides freshly coated with Norland optical adhesive 63, and flattened on the slides using the hand roller. Next, the slide was exposed to UV light for 8–10 s using a UV transilluminator (model GL-3120, Kylin-Bell, Haimen, Jiangsu, China). The tissue slides were transferred to dry ice, allowed to freeze for 5 min, then the adhesive tape was removed, and the slides were stored at −80°C until staining.

### Hematoxylin-Eosin and Sirius Red Staining

Slides were allowed to warm to room temperature, then stained with hematoxylin-eosin (HE) and Sirius Red as previously described (Liu et al., 2017; Huang et al., 2021). Sections were examined using a BX40 light microscope (Olympus, Feasterville, PA, United States) and photographed at magnifications of ×0.5–20.

### Multiplex Immunohistochemistry

Slides were allowed to warm to room temperature and fixed in 10% neutral-buffered formalin, then membranes were permeabilized using 0.25% Triton X-100. Endogenous peroxidase activity was quenched using 3% hydrogen peroxide. Slides were washed in 1 × TBST, then subjected to cyclic staining according to the manufacturer’s instructions (catalog no. NEL861001KT, Akoya Bioscience, Marlborough, MA, United States): after antigen retrieval, slides were blocked, incubated with primary antibody (Supplementary Table S2), incubated with horseradish peroxidase (HRP)-conjugated secondary antibody, stained with Opal, and stripped of primary and secondary antibodies. Slides were washed three times in 1 × TBST (2 min each) at room temperature with mild agitation, then nuclei were counterstained by dropping 200 μL of a DAPI working solution onto the slides. Slides were washed again with 1 × TBST at room temperature, then fluorescent anti-quencher was dropped onto the sample, and finally a coverslip was added. Slides were stored at 4°C and protected from light until subsequent scanning.

Multispectral images were acquired using the Vectra Polaris Automated Quantitative Pathology Imaging System. Multispectral image analysis of mIHC staining was performed using inForm Tissue Finder™ software (Version 2.3, PerkinElmer/Akoya, Marlborough, MA, United States). Thresholds for antibody positivity were calibrated for each slide individually. Next, a machine learning algorithm was trained to accurately segment background and cells based on user-specified annotations, where cells were defined based on nuclear DAPI signal and signal from epithelial markers. All images were reviewed after batch processing; tissue folds and other technical artefacts were excluded from further image analysis. On each slide, a “3 × 3” field of view (measuring 2,793 × 2094 μm, maximum area could be detected by inForm Tissue Finder™ software) was selected by experienced orthopedic researchers, who then counted the macrophages of different subtypes in the junction region within the field of view. In addition, two experienced orthopedic physicians selected fields of view at ×20 magnification on slides of Ficat stage III NONFH bone tissue, and they counted macrophages of different subtypes in perivascular and avascular areas.

### Cytometric Bead Array for the Detection of Macrophage Associated Cytokines

A cytometric bead array using the LEGEND 13-plex™ Human Macrophage/Microglia Panel (catalog no. 740503; Biolegend, San Diego, CA, United States) was performed to determine levels of macrophage-associated cytokines in plasma and synovial fluid. Briefly, 25 μL of sample, either plasma diluted 1:2 or synovial fluid diluted 1:5 in 1 × Wash Buffer, was incubated with microbeads, then with detection antibodies. Finally, samples were processed on a flow cytometer (CytoFLEX S, Beckman Coulter, Indianapolis, IN, United States), and data were analyzed using LEGEND plex™ Data Analysis Software.

### Immunohistochemistry

Slides of bone tissue were allowed to warm to room temperature, then fixed in 10% neutral-buffered formalin. Membranes in the tissue were permeabilized using 0.25% Triton X-100 (catalog no. 9002-93-1; Sigma-Aldrich, Darmstadt, Germany). Endogenous peroxidase activity was quenched using 3% hydrogen peroxide (Solarbio, Beijing, China), and antigens were retrieved using citrate buffer on a hot plate at 85°C. Non-specific reactivity on slides was blocked by incubating them in 5% bovine serum albumin at room temperature, then primary antibody (diluted 1:100) was added for overnight incubation at 4°C. Next, secondary antibody was applied for 60 min at room temperature. Slides were rinsed with 1 × Tris-buffered saline containing 0.5% Tween-20 (TBST), incubated for 30 min with avidin–biotin peroxidase (diluted 1:200), rinsed again in 1× TBST, then developed using 3,3-diaminobenzidine (Vector Laboratories, Burlingame, CA, United States). Slides were examined using an Olympus BX40 light microscope and photographed at ×20 magnification.

### Quantification and Statistical Analysis

Data were expressed as mean ± standard deviation (SD). Differences in cytokine levels, collagen content, or numbers of blood vessels were calculated across the three groups of patients with steroid-induced, alcohol-induced, or idiopathic ONFH, as well as the patients with osteoarthritis. Differences were assessed for significance using one-way analysis of variance (ANOVA). Pairwise comparisons of mean values were assessed for significance using Student’s t test. Statistical analysis was performed using GraphPad Prism 7.0 (San Diego, CA, United States), and differences associated with *p* < 0.05 were considered statistically significant. Sirius Red-stained images were semi-quantified using ImageJ. When appropriate, levels of significance were marked with * (*p* < 0.05), ** (*p* < 0.01) or *** (*p* < 0.001).

## Results

### Inflammatory Cells Were Enriched Mainly in the Reparative Area and Their Numbers Gradually Fell During Progressive-Stage to End-Stage Non-Traumatic Osteonecrosis of the Femoral Head Specimens

Chronic inflammation was previously identified as a remarkable feature of osteonecrosis (Goodman and Maruyama, 2020). The formation of a chronic inflammatory microenvironment in the local lesion is associated with enrichment of inflammatory cells. Thus, we collected the imaging information and harvested femoral heads from patients in different stages of human NONFH (Figure 1A). And then the large H&E sections were performed in femoral head (Figure 1B). In progressive stage (Ficat Stage III) and end-stage (Ficat Stage IV) NONFH, we discovered that inflammatory cells were mainly distributed in the reparative area while we could rarely detect inflammatory cells in the osteoarthritis femoral head (Figure 1C). In the reparative area, approximately 100 inflammatory cells per field were found in femoral heads from patients with Ficat stage III steroid-induced, alcohol-induced or idiopathic ONFH (Figure 1D), while the number of inflammatory cells per field fell to approximately 60 in Ficat stage IV (Figure 1E). The numbers of inflammatory cells fell from Ficat stage III to IV in patients with different types of ONFH. In the necrotic area, we did not observe notable infiltration of inflammatory cells (Figure 1F). Regardless of the type or stage of ONFH, and there was no difference in the numbers of inflammatory cells between patients with NONFH or osteoarthritis (Figures 1G,H). Our results suggest that inflammatory cells may play an important role in facilitating NONFH.

### Macrophage M1/M2 Imbalance Increased From Progressive-Stage to End-Stage Non-Traumatic Osteonecrosis of the Femoral Head Specimens

**FIGURE 2:**
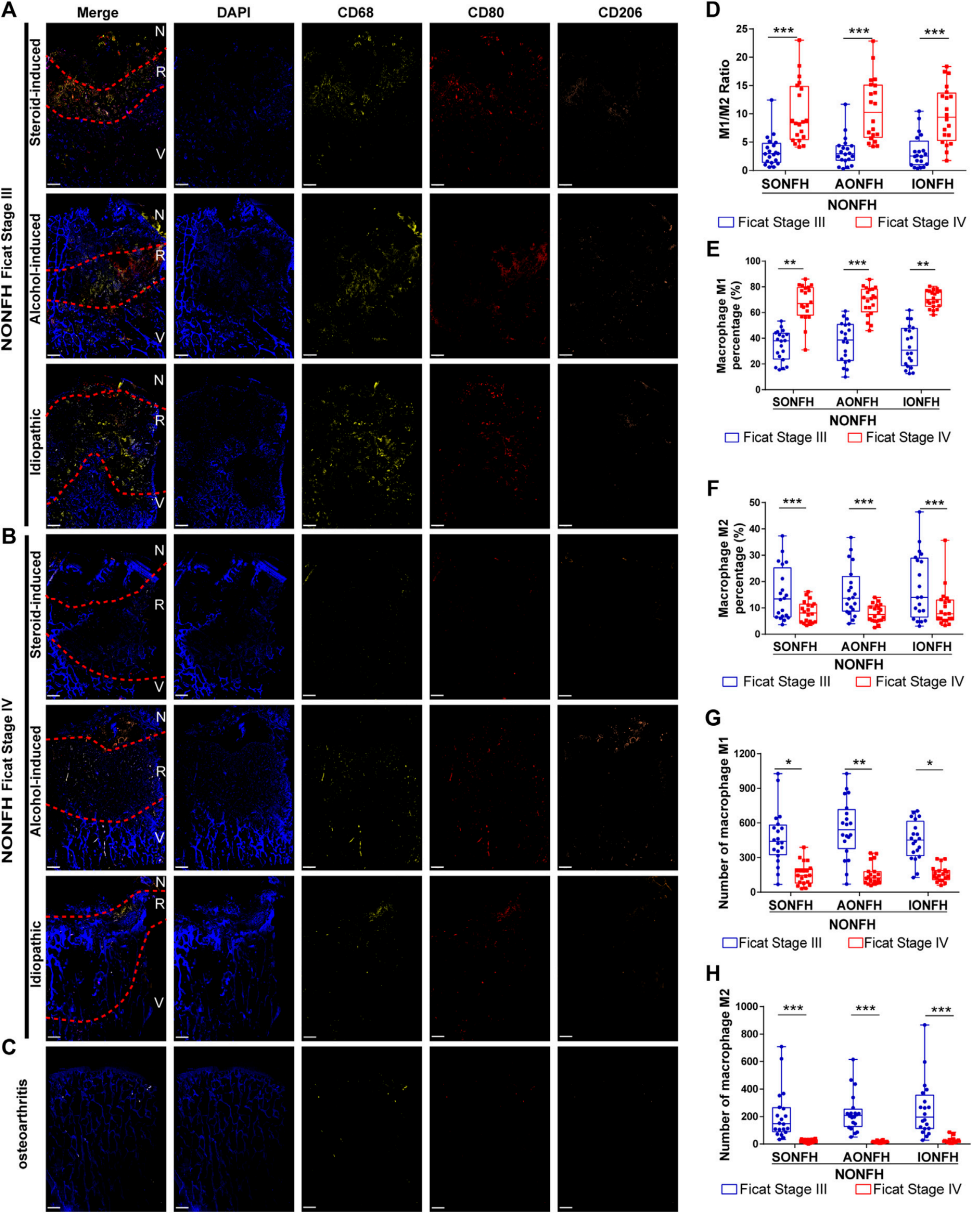
Macrophage M1/M2 imbalance increased from progressive-stage to end-stage NONFH **(A,B)** Representative mIHC images of M1 (CD68^+^CD80^+^CD206^-^) and M2 (CD68^+^CD80^−^CD206^+^) macrophages in sections from patients with different types NONFH in the **(A)** progressive stage or **(B)** end-stage. Scale bar, 2 μm **(C)** Representative mIHC images of M1 and M2 macrophages in patients with osteoarthritis. Scale bar, 2 μm **(D)** Comparison of M1/M2 macrophage ratios in patients with different types and stages of NONFH **(E,F)** Comparison of the percentages of **(E)** M1 and **(F)** M2 macrophages in patients with different types and stages of NONFH **(G,H)** Comparison of the numbers of **(G)** M1 and **(H)** M2 macrophages in patients with different types and stages of NONFH. *p < 0.05; **p < 0.01; ***p < 0.001.

Bone tissue injury or necrosis activates mainly innate immunity, in which macrophages are the main inflammatory cells. Initial activation of macrophages to adopt a pro-inflammatory M1 phenotype in order to establish acute inflammation, followed by a transition to anti-inflammatory M2 phenotype appears to be essential in the reconstruction and repair of tissues and organs. Given the difficulties of labeling M1 and M2 macrophages within bone tissue on the same slide, we adopted an innovative mIHC staining method to stain the hard tissue (femoral head) and label macrophages of different phenotypes. We discovered that M1 macrophages (CD68^+^CD80^+^CD206^-^) and M2 macrophages (CD68^+^CD80^−^CD206^+^) were enriched in the reparative area in sections from patients with different types and stages of NONFH (Figures 2A,B). In contrast, significant macrophage enrichment was not observed in sections from patients with osteoarthritis (Figure 2C). Previous work demonstrated that the macrophage M1/M2 ratio is >0.5 in synovial tissue from patients with chronic inflammatory bone diseases such as osteoarthritis or rheumatoid arthritis (Ma et al., 2021). We found that the M1/M2 ratio increased from 3 to 10 as patients with NONFH progressed from Ficat stage III to IV (Figure 2D). As disease progressed based on Ficat stage, the M1 percentage increased from 40 to 70% (Figure 2E), but the M2 percentage decreased from 15 to 7% (Figure 2F). However, interestingly, numbers of M1 macrophages (CD68^+^CD80^+^CD206^-^) and M2 macrophages (CD68^+^CD80^−^CD206^+^) fell during progression from Ficat stage III to IV (Figures 4G,H). These founds indicated that the macrophage M1/M2 imbalance participated in the NONFH.

### Relative Proportions of M1 and M2 Macrophages Were Opposite in Perivascular and Avascular Areas, and Numbers of Blood Vessels Increased in the Reparative Area During Progressive-Stage of Non-Traumatic Osteonecrosis of the Femoral Head Specimens

**FIGURE 3:**
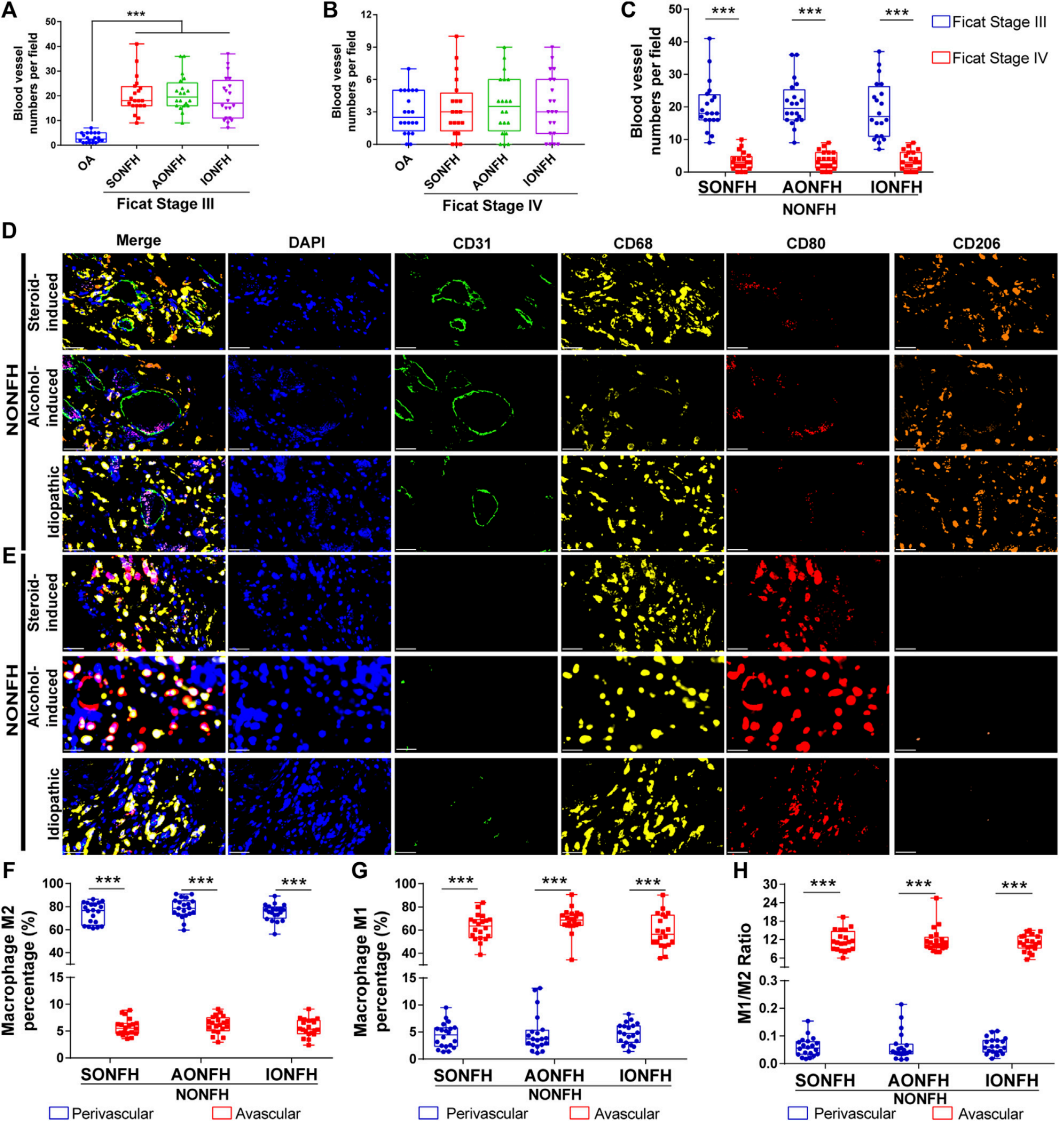
Relative proportions of M1 and M2 macrophages were opposite in perivascular and avascular areas, and numbers of blood vessels increased in the reparative area during progressive stage of NONFH **(A)** Comparison of numbers of blood vessels between patients with different types of progressive-stage NONFH or with osteoarthritis **(B)** Comparison of numbers of blood vessels between patients with different types of end-stage NONFH or with osteoarthritis **(C)** Comparison of numbers of blood vessels between patients with progressive-stage or end-stage NONFH **(D,E)** Representative mIHC images of M1 macrophages (CD68^+^CD80^+^CD206^-^) and M2 macrophages (CD68^+^CD80^−^CD206^+^) in perivascular areas (identified based on CD31^+^ vascular endothelial cells) and avascular areas in the reparative area in NONFH. Scale bar, 50 μm **(F,G)** Comparison of percentages of **(F)** M2 macrophages and **(G)** M1 macrophages in perivascular and avascular areas in patients with different types of progressive-stage NONFH **(H)** Comparison of macrophage M1/M2 ratios in perivascular and avascular areas in patients with different types of progressive-stage NONFH. *p < 0.05; **p < 0.01; ***p < 0.001.

NONFH used to be called “avascular necrosis of the femoral head”, highlighting the important role of blood vessels in the occurrence, development, regeneration and repair of NONFH. Nevertheless, the relationships between macrophages and blood vessels in NONFH remain unclear. In a 2,793 μm × 2094 μm detection field, the number of blood vessels (defined as ring structures formed by CD31^+^ cells) increased to approximately 20 in patients with Ficat stage III (progressive Stage) NONFH (Figure 3A). Fewer than five blood vessels per field were detected in patients with Ficat stage IV (end-stage) disease, similar to osteoarthritis patients (Figure 3B). Among patients with different types and stages of NONFH, the number of blood vessels gradually decreased as the disease progressed from progressive-stage to end-stage (Figure 3C). Furthermore, among patients with progressive stage NONFH, M2 macrophages were the main subtype in the perivascular area (Figure 3D), and their proportion reached nearly 80% (Figure 3F), while the proportion of M1 macrophages was less than 5% (Figure 3G). Conversely, M1 macrophages were the dominant subtype in avascular areas (Figure 3E), accounting for over 60% of all macrophages (Figure 3G), while only 5% of macrophages were M2 (Figure 3F). The ratio of M1/M2 macrophages was 0.05 in perivascular areas and 13 in avascular areas (Figure 3H). These results suggest that as NONFH progresses from progressive-stage to end-stage, the numbers of blood vessels in the reparative area fall and the M1/M2 ratio increases. These observations imply that macrophage M1/M2 imbalance facilitates the progression of NONFH.

### IL-6 and IL-1β Maintained the Local Chronic Inflammatory Microenvironment in Human Non-Traumatic Osteonecrosis of the Femoral Head Specimens

**FIGURE 4:**
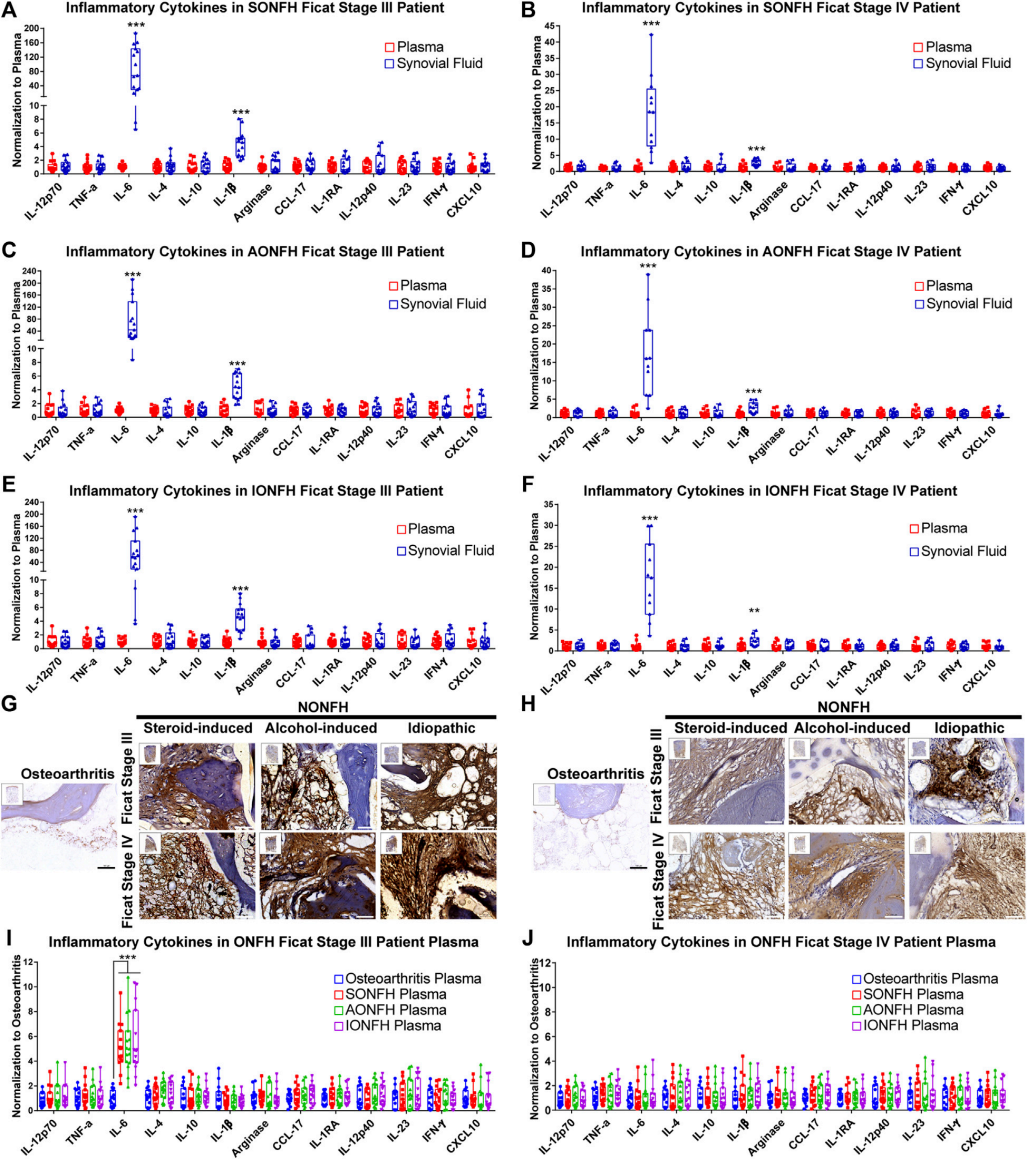
IL-6 and IL-1β maintained the local chronic inflammatory microenvironment in human NONFH **(A-F)** Comparison of the levels of cytokines in synovial fluid and plasma in patients with different types and stages of NONFH **(G,H)** Representative images of **(G)** interleukin-6 (IL-6) and **(H)** interleukin-1β (IL-1β) in femoral head specimens from patients with different types and stages of NONFH or with osteoarthritis, based on immunohistochemistry (IHC). Scale bar, 100 μm **(I,J)** Comparison of cytokine levels in plasma of patients with different types and stages of NONFH or with osteoarthritis. *p < 0.05; **p < 0.01; ***p < 0.001.

Macrophages are the vital modulators of inflammation in immune responses, and they participate in chronic inflammation, which was previously identified as a remarkable feature of osteonecrosis (Goodman and Maruyama, 2020). However, which inflammatory cytokines are involved in maintaining chronic inflammation and what dynamic changes they show are unclear. In our studies, we innovatively used the innovative cytometric bead array to detect cytokines in synovial fluid and plasma in patients with different types and stages of ONFH. We found that levels of IL-6 and IL-1β were significantly higher in synovial fluid than in plasma in progressive-stage and end-stage patients (Figures 4A–F.) In progressive stage NONFH, amounts of IL-6 and I-1β in synovial fluid were >50 or four times the amounts in blood, respectively (Figures 4A,C,E). However, when the disease progressed to end-stage, the levels of IL-6 and IL-1β in synovia fluid dropped to about 15 or two times the levels in plasma, respectively (Figures 4B,D,F). Simultaneously, histology showed high expression of IL-6 and IL-1β in the femoral head of NONFH patients (Figures 4G,H). These results suggest that IL-6 and IL-1β are the main inflammatory cytokines in the local lesion and that they maintain the chronic inflammatory microenvironment.

Given the observed enrichment of IL-6 and IL-1β in local lesions, we measured their levels in the circulation of NONFH and osteoarthritis patients. Levels of IL-6 were nearly five times in progressive stage NONFH plasma than in osteoarthritis, while we did not observe such a difference between end-stage NONFH and osteoarthritis patients (Figures 4I,J). These results suggest that the local lesion may secrete IL-6 into the plasma during progressive stage NONFH.

### Fibrous Tissue Formed and Hindered the Repair of Necrotic Areas in Non-Traumatic Osteonecrosis of the Femoral Head Specimens

**FIGURE 5:**
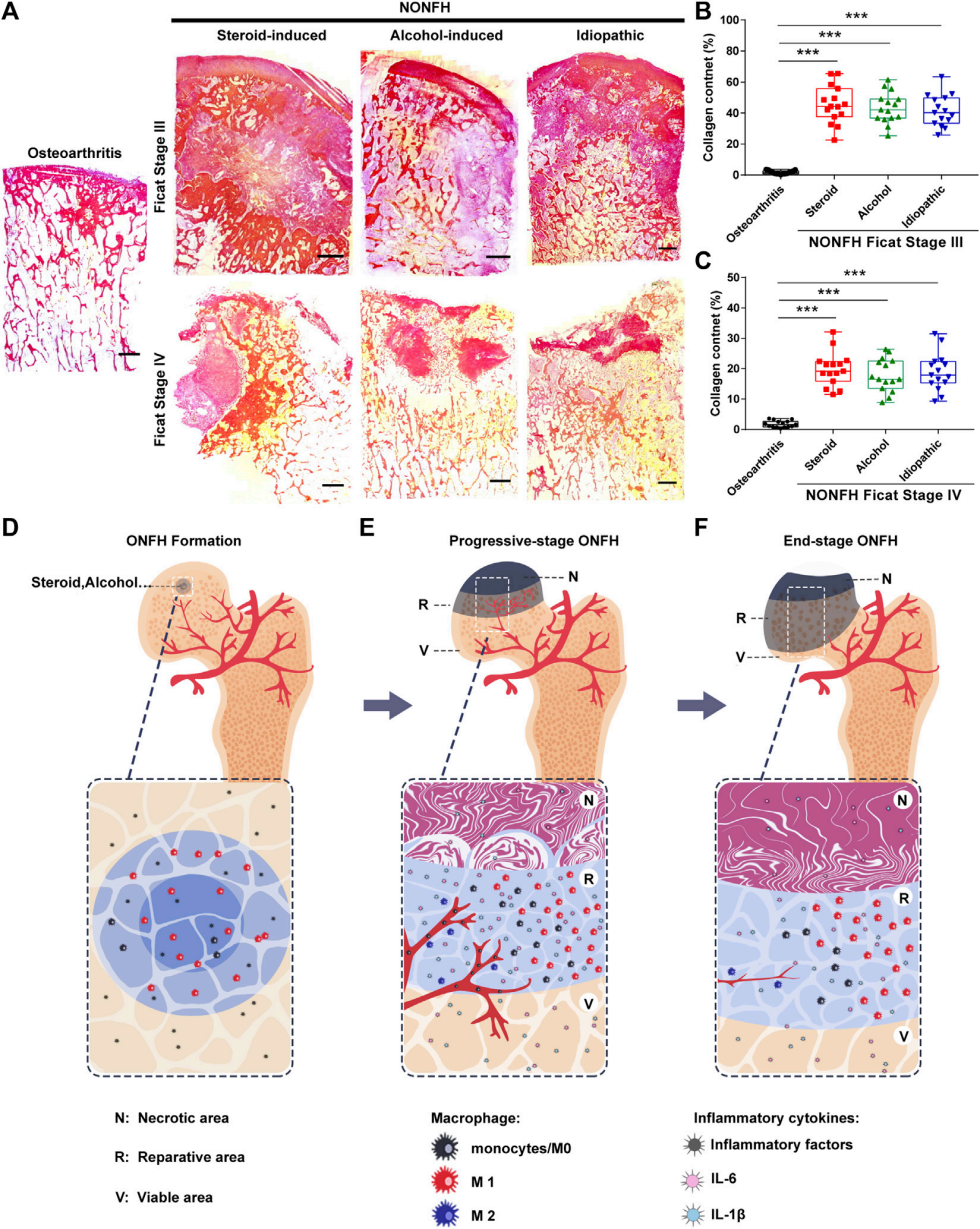
Fibrous tissue formed and hindered the repair of necrotic areas in NONFH **(A)** Representative images of Sirius Red-stained sections from patients with different types and stages of NONFH. Scale bar, 2.5 mm **(B,C)** Collagen quantification in sections from patients with different types NONFH in Ficat Stage III **(B)** or Ficat stage IV **(C)**, based on Sirius red staining **(D–F)** Schematic showing how macrophage M1/M2 imbalance participates in the formation of chronic inflammation and tissue fibrosis, and facilitates the progression of human NONFH: macrophages migrate to the local bone lesion and adopt the M1 phenotype, then secrete abundant IL-6, IL-1β and other inflammatory factors after bone necrotic **(D)**. Those cytokines induce blood vessels formation and recruit monocytes into the local lesion. And then the monocyte/macrophages are polarized to M1 (avascular regions) and M2 (around blood vessel) subtype. The M1 subtype is dominating in local lesion and secreting the inflammatory cytokines to formation the inflammatory microenvironment **(E)**. When osteonecrosis progresses to the base of the femoral head (end-stage), inflammation subsided, the number of macrophages M1 decreased, bone necrotic with bone formation **(F)**.

Tissue fibrosis is the result of chronic inflammation, which hinders tissue regeneration and repair. Although tissue fibrosis has been confirmed to exist in local lesions in NONFH, its dynamic changes have not yet been clarified. Thus, we performed Sirius Red staining and found that the amount of fibrous tissue in the necrotic area decreased from progressive-stage to end-stage NONFH (Figure 5A). In progressive stage NONFH, over 40% of the field was occupied by fibrous tissue (Figure 5B), but this proportion fell to 20% in end-stage disease (Figure 5C). This decline appeared to be an artifact, however, reflecting loss of cartilage and necrotic tissue during specimen preparation and decalcification. Specimens from patients with end-stage disease lacked cartilage and subchondral bone and were closer to the base of the femoral neck than the specimens from patients in progressive stage. Therefore, fibrosis was actually more prominent in end-stage NONFH than in progressive stage.

## Discussion

In this study, we innovatively applied mIHC to femoral head specimens (hard tissues) and found that as NONFH progression from progressive stage to end-stage, macrophage infiltration in the reparative area as well as macrophage M1/M2 imbalance increased, while the number of blood vessels declined. More interestingly, we discovered that in the reparative area in progressive stage NONFH, M2 macrophages were distributed mainly in perivascular area, while M1 macrophages were enriched in avascular areas. Furthermore, we performed a cytometric bead array in synovial fluid and plasma. We found that IL-6 and IL-1β were the dominant inflammatory cytokines, and they maintained the chronic inflammatory microenvironment in the local lesion. More importantly, IL-6 was transferred from the local lesion into the circulation and could be used as a hematological marker of the progressive stage NONFH patients. Moreover, we found that abundant fibrosis tissue formed in the necrotic area and hindered the reconstruction of bone structure in NONFH.

### Macrophage in Non-Traumatic Osteonecrosis of the Femoral Head Specimens

Although bone injury and reconstruction were once thought to involve only the osteoblasts derived from mesenchymal stem cells and osteoclasts derived from monocytes, recent research has highlighted more complex interactions between bone and immune cells (Loi et al., 2016; Tsukasaki and Takayanagi, 2019). Macrophages are the sentinels of innate immunity, they are responsible for the removal of apoptotic or necroptotic cell debris, and they initiate inflammatory response (Vergadi et al., 2017). The heterogeneity and plasticity of macrophages allow them to exert complex immunomodulatory functions. However, the relative amounts of different cells and subtypes, and their expression of different proteins, have not yet been analyzed in hard tissue. Considering the plasticity and diversity of macrophages, we first applied mIHC to large pathological sections of hard tissues (femoral head). As a result, we were able to detect and quantify different subtypes of macrophages and vascular endothelial cells in various areas of bone tissue affected by NONFH. Multispectral image analysis of the necrosis femoral head revealed that macrophages accumulate in the reparative area, which are therefore likely to be their “primary battlefield”. We also found that the macrophage M1/M2 ratio gradually increased from progressive-stage to end-stage NONFH. Similarly, the M1/M2 macrophage ratio increased during progressive-stage of medication-related human osteonecrosis of the jaw, based on double immunofluorescence to detect M1 macrophages (CD68^+^ iNOS^+^) and M2 macrophages (CD68^+^ CD206^+^), but the absolute numbers of M1 and M2 macrophages decreased from progressive to end-stage disease (Paschalidi et al., 2021). Consistent with this result, we also found that the total number of M1 and M2 macrophages decreased from the progressive stage to end-stage of NONFH (Paschalidi et al., 2021). Interestingly, NONFH used to be called “avascular necrosis of the femoral head”, and previous work has demonstrated that the number of blood vessels decreases as the disease progresses to end-stage (Zhao et al., 2020). Although blood vessels are considered a prerequisite for tissue repair, they may contribute to pathology during the progression of NONFH. The macrophages that we observed to accumulate in the reparative area likely arose from monocytes that migrated from the neovascularization. Blood vessels transport not only monocytes but also oxygen and nutrients, which can lead macrophages to adopt and maintain an M2 phenotype (Sica and Mantovani, 2012; Mehla and Singh, 2019). Thus, we investigated the completely different distributions of M1 and M2 macrophages in perivascular and avascular in the reparative area in NONFH. Our findings suggest that the neovascularization and M2 macrophages in the reparative area are insufficient to reconstruct normal bone structure in NONFH, consistent with the poor prognosis of patients with NONFH.

### Chronical Inflammatory Microenvironment (IL-6 and IL-1β) in Non-Traumatic Osteonecrosis of the Femoral Head Specimens

Increasing evidence suggests that macrophage M1/M2 imbalance facilitates the formation of a chronic inflammatory microenvironment. Pro-inflammatory M1 macrophages are the main producers of inflammatory cytokines (e.g., IL-6, IL-1β) in a chronic inflammatory microenvironment (Toshio Tanaka et al., 2014; Dinarello, 2019; Zhang et al., 2021). In our study, we observed that levels of IL-6 and IL-1β dramatically and persistently increased in local lesions of patients with progressive-stage and end-stage NONFH. Levels of IL-6 in circulation were also elevated in progressive-stage NONFH patients than in osteoarthritis. Intriguingly, IL-6 acts as a “double-edged sword”, depending on whether it acts during acute or chronic inflammation (Gabay, 2006; Toshio Tanaka et al., 2014). During the acute inflammatory response in the early phase of tissue injury or infection, IL-6 promotes the removal of damaged tissue or infected cells (Gabay, 2006; Toshio Tanaka et al., 2014). Persistent dysregulation of IL-6, however, creates a chronic inflammatory microenvironment leading to pathology (Toshio Tanaka et al., 2014; Kang and Kishimoto, 2021; Villar-Fincheira et al., 2021). IL-6 can activate the Rank signaling pathway to promote the formation of osteoclasts, thereby inhibiting osteogenesis and perturbing bone metabolism (Terpos et al., 2011; Kim et al., 2016; Zhu et al., 2018; Llorente et al., 2020). Inhibiting or knocking out IL-6 in human patients or animal models of chronic inflammatory bone disease limits bone loss and slows disease progression (Keisuke Tanaka et al., 2014; Kim et al., 2016; Zhu et al., 2018; Paschalidi et al., 2021). Interestingly, our results are similar to those reported for patients with drug-induced osteonecrosis, who show increased IL-6 levels in the local lesion and/or in the circulation (Bagan et al., 2014; Badros et al., 2021; Paschalidi et al., 2021). In the piglet model of ischemic ONFH, IL-6 inhibitors inhibit osteoclastogenesis and delay progression of osteonecrosis (Ren et al., 2021), further highlighting the important role of IL-6 in the chronic inflammation of osteonecrosis. The increased accumulation of IL-6 at the site of injury most likely leads to increased levels in the circulation: indeed, the level of IL-6 in plasma was over five times in progressive-stage NONFH than in osteoarthritis.

In contrast to IL-6, IL-1β showed weaker upregulation in local lesions in our patients with NONFH. IL-1β exerts pro-inflammatory effects during acute and chronic inflammation (Dinarello, 2018). Activation of IL-1β signaling pathways promotes apoptosis, inhibits osteogenesis, activates osteoclasts, induces bone resorption and accelerates the progression of chronic inflammatory bone diseases such as rheumatoid arthritis, osteoporosis, and periodontitis (Ruscitti et al., 2015; Cheng et al., 2020; Maruotti et al., 2020). In fact, IL-1β inhibition is considered a promising strategy for inhibiting the progression of chronic inflammatory diseases (Liberale et al., 2019). IL-6 and IL-1β may jointly influence not only over bone metabolism, but also angiogenesis and tissue fibrosis (Zhang et al., 2021). Both cytokines recruit endothelial cells, fibroblasts and other tissue-regenerating cells to sites of injury, where they induce the secretion of VEGF and PDGF, which in turn promote formation of blood vessels and production of collagen. This expands the extracellular matrix and leads to the fibrosis characteristic of chronic inflammatory bone disease (Toshio Tanaka et al., 2014; Dinarello, 2019; Zhang et al., 2021). Our patients with different types of ONFH showed extensive fibrosis in the progressive-stage and end-stage, whereas blood vessel formation was observed only in the progressive-stage, similar to analyses of intraosseous blood vessels by digital subtraction angiograph and Micro-computed tomography (Zhao et al., 2020). These results indicate a strong repair response during progressive-stage NONFH. IL-6 has also been suggested as an essential cytokine in the pathogenesis of chronic inflammatory disease (Grom et al., 2016). Otherwise, IL-6 may also promote IL-1β secretion by macrophages, synovial fibroblasts and other cells (Fernando et al., 2014; Symons et al., 2021). Based on these considerations, we believe that IL-6 may contribute more than IL-1β to angiogenesis, fibrosis formation and bone resorption during the progression of NONFH. Our results suggest that IL-6 and IL-1β play a vital role in the formation of a chronic inflammatory microenvironment, angiogenesis and fibrosis during the progression of NONFH.

### Study Limitation

In this study, our team only collected and analysis progressive-stage and end-stage clinical specimens in NONFH due to the occult nature of early NONFH. At the same time, osteoarthritis patients’ clinical specimens as a control may affect the result, femoral head and blood samples from amputated or cadaveric patients should be used in future studies. In the detection of macrophage, the multiplex immunohistochemistry were the only means performed in our study. We also tried flow cytometry to detect different macrophage phenotypes, but it was difficult to extract cells from the necrotic femoral head and the low viability of the extracted cells limited the application. Otherwise, due to the large size of necrotic bone specimens, we can only select typical areas (2,793 Non-traumatic Osteonecrosis of the Femoral Head Specimens × 2094 μm per area) in each sample for analysis which was limited by the software. Finally, our study is based on a small sample size, and more large studies are needed to confirm the role of macrophages and chronic inflammation in human NONFH.

## Conclusion

In summary, our work suggests that the increasing macrophage M1/M2 imbalance and declining number of blood vessels facilitate the progression of NONFH. We also demonstrated that NONFH is a chronic inflammatory disease involving persistent enrichment with IL-6 and IL-1β, osteonecrosis and fibrosis in the local lesion. Inhibiting inflammation, promoting the resolution of inflammation, switching macrophages to an M2 phenotype, or inhibiting their adoption of an M1 phenotype may be useful therapeutic strategies against NONFH.

## Data Availability

The raw data supporting the conclusions of this article will be made available by the authors, without undue reservation.
