# Supplementary material for: The Dynamic Feature of Macrophage M1/M2 Imbalance Facilitates the Progression of Non-Traumatic Osteonecrosis of the Femoral Head
Source: Front Bioeng Biotechnol. 2022 Apr 27;10:912133. doi: 10.3389/fbioe.2022.912133 (PMC9094367; doi:10.3389/fbioe.2022.912133)
Supplement: Supplementary file 1 [file DataSheet1.PDF]

Supplementary Material

Supplementary Table 1

Demographic data of NONFH and osteoarthritis patients.

Variable	SONFH (Ficat Stage III + IV)	AONFH (Ficat Stage III + IV)	IONFH (Ficat Stage III + IV)	*P* (Among ONFH)	Osteoarthritis
N	30	30	30	-	15
Age	38.6 ± 9.6	42.5 ± 11.6	41.5 ± 13.1	.400	62.6 ± 8.7
Gender (Female/Male)	18/12	8/22	10/20	.020	10/5
BMI	28.68 ± 5.98	26.52 ± 6.21	25.45 ± 5.79	.111	28.68 ± 5.98
Time of Diagnosis (Month)	14.2 ± 6.5	15.1 ± 7.4	13.7 ± 4.6	.683	124.6 ± 32.6
Comorbidities (N)	12	10	8	.549	9
Smoking or past smoking (N)	14	20	18	.279	6
Previous hip surgery	0	0	0	-	0
Bisphosphonate therapy	0	0	0	-	0

BMI: Bone Mass Index; NONFH: Non-traumatic osteonecrosis of the femoral head; SONFH: Steroid-induced osteonecrosis of the femoral head; AONFH: Alcohol-induced osteonecrosis of the

femoral head; IONFH: Idiopathic osteonecrosis of the femoral head; ONFH: osteonecrosis of the femoral head.

Supplementary Table 2

Multiplex immunohistochemistry (mIHC) and immunohistochemistry antibodies used in this study

Application	Primary antibody	Dilution	Ref	Provider	Secondary antibody
mIHC	CD68	1:1000	ab201340	Abcam	-
mIHC	CD80	1:1000	AMB140	R&D	-
mIHC	CD206	1:1000	ab64693	Abcam	-
mIHC	CD31	1:1000	3528s	CST	-
IHC	IL-1β	1:1000	12242s	CST	Anti-Mouse HRP
IHC	IL-6	1:1000	Ab6672	Abcam	Anti-Rabbit HRP
